# Association between blood metabolites and basal cell carcinoma risk: a two-sample Mendelian randomization study

**DOI:** 10.3389/fendo.2024.1413777

**Published:** 2024-07-09

**Authors:** Bingliang Wu, FuQiang Pan, QiaoQi Wang, Qian Liang, HouHuang Qiu, SiYuan Zhou, Xiang Zhou

**Affiliations:** ^1^ Department of Medical Cosmetology, The Second Affiliated Hospital of Guangxi Medical University, Nanning, Guangxi, China; ^2^ Department of Health Examination Center, The Second Affiliated Hospital of Guangxi Medical University, Nanning, Guangxi, China

**Keywords:** genome-wide association studies, Mendelian randomization, circulating metabolites, basal cell carcinoma, unsaturated fatty acids

## Abstract

**Background:**

Circulating metabolites, which play a crucial role in our health, have been reported to be disordered in basal cell carcinoma (BCC). Despite these findings, evidence is still lacking to determine whether these metabolites directly promote or prevent BCC’s progression. Therefore, our study aims to examine the potential effects of circulating metabolites on BCC progression.

**Material and methods:**

We conducted a two−sample Mendelian randomization (MR) analysis using data from two separate genome-wide association studies (GWAS). The primary study included data for 123 blood metabolites from a GWAS with 25,000 Finnish individuals, while the secondary study had data for 249 blood metabolites from a GWAS with 114,000 UK Biobank participants.GWAS data for BCC were obtained from the UK Biobank for the primary analysis and the FinnGen consortium for the secondary analysis. Sensitivity analyses were performed to assess heterogeneity and pleiotropy.

**Results:**

In the primary analysis, significant causal relationships were found between six metabolic traits and BCC with the inverse variance weighted (IVW) method after multiple testing [P < 4 × 10−4 (0.05/123)]. Four metabolic traits were discovered to be significantly linked with BCC in the secondary analysis, with a significance level of P < 2 × 10−4 (0.05/249). We found that all the significant traits are linked to Polyunsaturated Fatty Acids (PUFAs) and their degree of unsaturation.

**Conclusion:**

Our research has revealed a direct link between the susceptibility of BCC and Polyunsaturated Fatty Acids and their degree of unsaturation. This discovery implies screening and prevention of BCC.

## Introduction

Basal cell carcinoma (BCC) is the most common malignant tumor worldwide, with its incidence continuing to rise annually. Reports indicate that BCC incidence in the United States increases by 4% to 8% each year, and the World Health Organization projects nearly 1.2 million new BCC cases globally in 2020 (1, 2). BCC primarily affects individuals with Fitzpatrick skin types I and II. BCC risk factors include light eye color, freckles, and blonde or red hair. The most significant environmental risk factor is UV radiation exposure. Additionally, other potential risk factors include exposure to arsenic and other carcinogens, ionizing radiation, photosensitizing drugs, and chronic immunosuppression. BCC considerably impacts patients’ well-being and creates financial strain for affected individuals and society (2, 3). However, it remains unclear whether lifestyle and metabolic characteristics contribute to the development of BCC. Given the uncertain influence of daily diet and metabolic traits on BCC, investigating the relationship between metabolites and BCC is crucial.

In recent decades, mounting evidence has highlighted the crucial role of metabolic reprogramming and energy metabolism in cancer cell proliferation and metastasis (4, 5). Alterations in normal cell metabolism can promote cell growth and affect cell differentiation, rendering them more susceptible to cancer development (5). Moreover, targeted modulation of metabolites holds promise for cancer treatment by increasing the sensitivity of cancer cells to therapy (6, 7). Therefore, investigating the metabolites associated with BCC can aid in early detection and prevention of BCC, as well as provide insights into the underlying biological processes for effective treatment. Previous research has highlighted the impact of specific blood metabolites, such as prostaglandins and leukotrienes, on skin cancer development by influencing the inflammatory response. Additionally, associations have been found between BCC and the human prothrombin complex, 25-hydroxyvitamin D, vitamin D, and PUFAs (8–11). Consequently, serum metabolites likely play a crucial role in BCC occurrence and progression. However, conducting randomized controlled trials (RCTs) to investigate this relationship is challenging due to ethical considerations and limitations inherent in observational studies, such as susceptibility to confounding factors and reverse causality. Novel methods that minimize the impact of confounding factors are needed to advance our understanding.

The Mendelian randomization (MR) approach utilizes genetic variants as proxies for exposure variables to investigate their effect on particular outcomes (12). Due to the random allocation of single-nucleotide polymorphisms (SNPs) at conception, they are less susceptible to confounding influences, while reverse causality bias is reduced because genetic markers do not affect outcomes (13). This makes MR analysis an excellent method for exploring the relationship between metabolites and BCC. Therefore, we employ several MR techniques in this article and integrate extensive human genomic datasets to estimate the potential causal effects between metabolites and BCC.

## Materials and methods

### Study design

**Figure 1:**
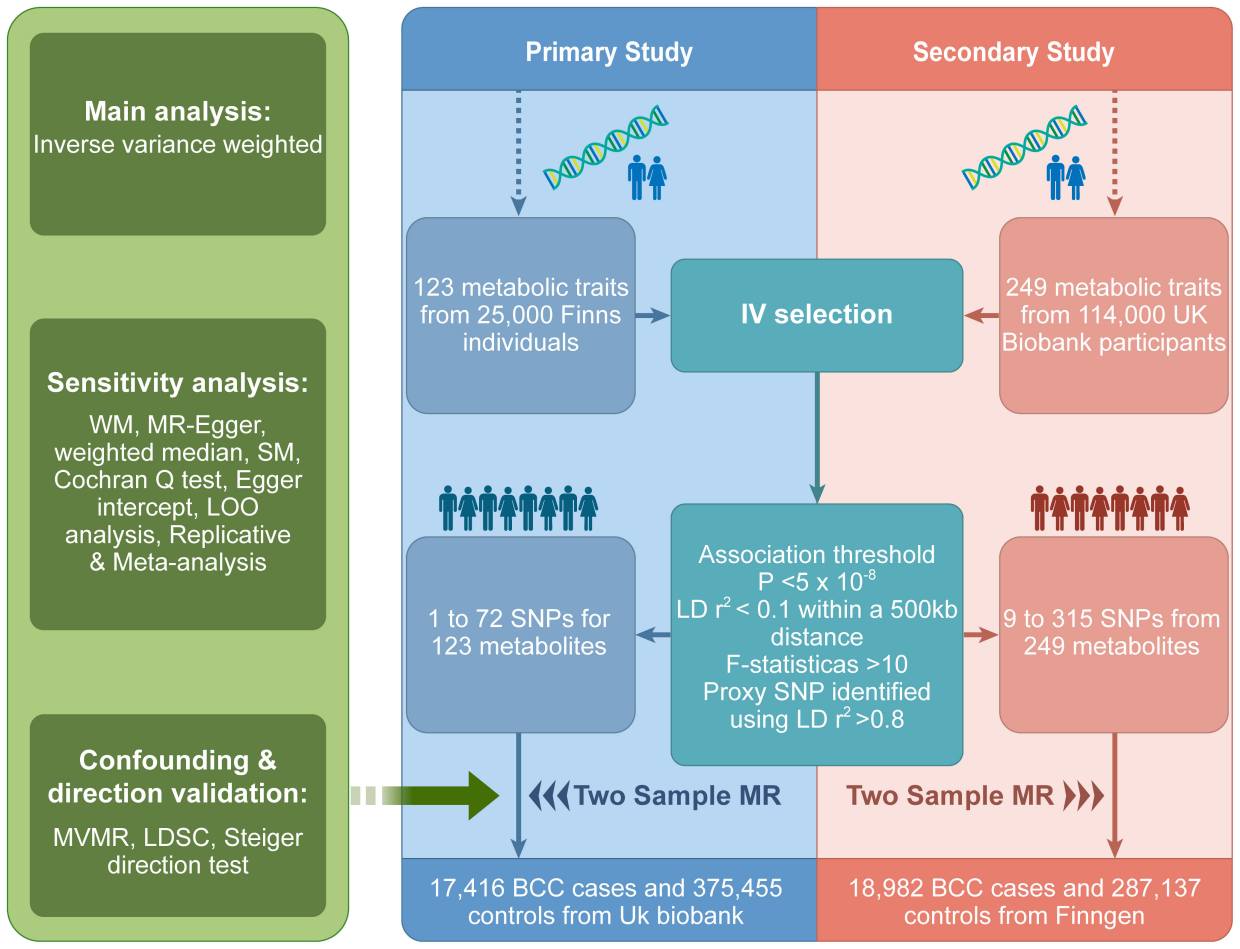
MR study overview. MR, Mendelian randomization; IV, instrumental variables; SNPs, single nucleotide polymorphisms; LD, linkage disequilibrium; WM, weighted mode; LOO, leave one out; SM, simple mode; BCC, Basal cell carcinoma; MVMR, Multivariate Mendelian randomization.

The design of the study is illustrated in **Figure 1**. We utilized instrumental variables derived from two distinct metabolomics datasets for our primary and secondary analyses. Notably, the datasets we chose include a variety of similar metabolites, allowing for cross-validation between our two discovery cohorts. This approach has been similarly employed in other studies (14, 15). The central focus of our research was to scrutinize the causal influence of genetically predicted serum metabolite concentrations on BCC susceptibility. Our Two-Sample MR analysis was designed with variables that meet the criteria for credible genetic instrumental variables. We conducted a thorough evaluation of blood metabolites for their potential causal effects on BCC using several criteria: (1) A significant p-value in the primary analysis (IVW, p < 0.05); (2) Consistency in direction and magnitude across the three MR methods; (3) Absence of heterogeneity or horizontal pleiotropy in the MR results; (4) MR estimates were not significantly influenced by a single SNP (16). This approach reflects our adherence to the three key assumptions of MR analysis: Assumption 1, a robust association between genetic variants and exposure; Assumption 2, no association between genetic variants and confounding variables; Assumption 3, no influence of genetic variants on outcomes outside the specific exposure. Notably, this analysis used the STROBE-MR checklist for reporting MR studies (17).

### Metabolic profile for primary analyses

The primary analysis of metabolic profiles was conducted using data from a large, diverse cohort of 24,925 individuals from Finland, the Netherlands, and Estonia, as reported in the 2016 study by Kettunen et al. published in Nature Genetics (18). The dataset includes information on 123 different metabolites currently circulating in the body, such as specific lipids from lipoprotein subclasses, amino acids, fatty acids, and glycoproteins related to inflammation, among other measurements. Metabolite concentrations were assessed using high-throughput NMR spectroscopy. Data from each cohort were analyzed separately using an additive model and then combined through a fixed-effect meta-analysis, incorporating up to 12,133,295 SNPs. All metabolite concentrations were adjusted for age, sex, time since the last meal and the first ten principal components. The study findings are accessible through the Integrative Epidemiology Unit (IEU) OpenGWAS project.

### Metabolic profile for secondary analyses

The GWAS datasets from the Phase 1 release of the Nightingale Health Metabolic Biomarkers study were used for a secondary analysis of 249 human metabolite measurements. This study encompassed a selection of 115,078 participants from the UK Biobank cohort. The participants provided baseline plasma samples in a non-fasting state, preserved in EDTA, which were then subjected to metabolic profiling through high-throughput NMR technology. The profiling generated data on 168 metabolites presented in absolute concentrations (mmol/L) and 81 metabolite ratios (https://biobank.ndph.ox.ac.uk/ukb/label.cgi?id=220, accessed February 1, 2024). The panel included metrics on the metabolism of triglycerides and cholesterol, diverse fatty acid profiles, and various low-molecular-weight metabolites such as amino acids, ketones, and glycolysis-related substances. It offered differentiated measurements for triglycerides, phospholipids, total cholesterol, cholesterol esters, free cholesterol, and total lipid concentration across 14 distinct lipoprotein subclasses. For population structure considerations within the UK Biobank, the BOLT-LMM (linear mixed model) was employed, with further adjustments for variables such as age, sex, the interval since the last meal or drink, and the genotyping chip used (UKBB Axiom array or UK BiLEVE array). The full set of summary statistics is available on the IEU OpenGWAS project database.

### Basal cell carcinoma

The GWAS summary data for BCC in Europeans (GWAS ID ebi-a-GCST90013410, consisting of 17,416 cases and 375,455 controls) was acquired from the IEU Open GWAS database. The primary data source is the UK biobank, released in a 2021 publication in Nature Genetics by Adolphe C et al. (19). This is currently the largest GWAS dataset on BCC available in the IEU database. We used it as the outcome group data for our primary analysis to avoid population sample overlap. Our secondary analysis used BCC data from FinnGen (18,982 cases and 287,137 controls) as the outcome variable (20). This allows our exposure and outcome data to avoid population overlap, further strengthening our research’s credibility. We used data from two independent studies for replication analysis and meta-analysis to validate our results further. Both datasets can be obtained from the GWAS catalog (https://www.ebi.ac.uk/gwas/). The first dataset was published by Jiang L et al. in Nature Genetics in 2021, including 456,276 individuals from the British European population (GWAS catalog ID: GCST90041916) (21). The second dataset was published by Seviiri M et al. in Nature Communications in 2022, including 307,684 individuals from European populations in the United States, the United Kingdom, and Australia (GWAS catalog ID: GCST90137411) (22) (**Table 1**).

**Table 1:** Information on the sources of the data included.

Traits	Sample size	Year	Population	PMID	Web source
123 Circulating metabolites (primary analyses)	24,925	2016	European	27005778	https://doi.org/10.1038/ncomms11122
249 Circulating metabolites (secondary analyses)	115,078	2020	European	35692035	https://europepmc.org/article/MED/35692035
basal cell carcinoma (Adolphe C)	392,871	2021	European	33549134	https://doi.org/10.1186/s13073–021-00827–9
basal cell carcinoma (FinnGen)	306,119	2023	European	NA	https://r9.risteys.finngen.fi/
basal cell carcinoma (Seviiri M)	307,684	2022	European	36496446	https://doi.org/10.1038/s41467–022-35345–8
basal cell carcinoma (Jiang L)	456,276	2021	European	34737426	https://doi.org/10.1038/s41588–021-00954–4

### IV selection

We followed several steps to select genetic variants related to metabolites. These SNPs were selected based on a stringent genome-wide significance threshold (p < 5×10^−8^). We specifically chose these SNPs due to their low linkage disequilibrium (LD) with other SNPs (r2 < 0.1, clump window 500 kb), which ensured their role as independent instrumental variants for the corresponding metabolites. This strategy was common in previous serum metabolite MR studies (23–26). Next, we calculated F statistics for each SNP to evaluate their statistical significance. To guarantee sufficient variance for the metabolites, we discarded SNPs with an F value less than 10, considering them as inadequate instruments (27, 28). Then, we aligned the SNPs for both exposure and outcome. We used the allele frequency data for palindromic SNPs to infer the forward-strand alleles. Lastly, we did MR analysis on metabolites with more than two SNPs. We used proxies in high LD (r² > 0.8) from the European reference panel of the 1000 Genomes Project for SNPs missing in the outcome. We discarded those without proxies.

### LDSC and Steiger

To examine if the causal links we found were affected by common genetic factors, we used LDSC to measure the genetic correlation between BCC and the metabolites we identified. MR can often produce false positives when there is a genetic correlation between traits (29, 30). We also used the Steiger test to check if the causalities we observed were distorted by reverse causation. The Steiger test can tell us if the SNPs we included accounted for more of the BCC variation than the metabolites (31). If the SNPs had a higher contribution to the BCC risk than the metabolites (Steiger P > 0.05), it would suggest that the causal direction might be wrong.

### Replication and meta-analysis

We conducted another IVW analysis with two separate BCC GWAS datasets from the aforementioned GWAS Catalog consortium to ensure the validity of the metabolites we had selected. A subsequent meta-analysis was carried out to ascertain the definitive metabolites causally linked to BCC.

### Multivariable Mendelian randomization analysis

We used MVMR to calculate the direct influence of multiple exposures on a specific outcome while also adjusting for other exposures in this study. MVMR analysis allowed us to assess the causal connections between potential metabolites/ratio index and the risk of BCC, taking into account various common BCC risk factors (32).

### Statistical analyses

Using IVW analysis, we assessed the causal impact of blood metabolites on BCC. Depending on the presence of heterogeneity in the MR analysis, we applied either the fixed-effects or random-effects IVW model. If the random-effects analysis shows statistical evidence of causal effects, it indicates consistent support for the causal influence of exposure on the outcome, taking into account the heterogeneity of causal estimates due to variation specificity (33, 34). We also conducted a sensitivity analysis to assess any potential bias in the MR assumptions. We used a range of MR models, including MR-Egger regression, weighted median, simple mode, and weighted mode (WM), as additional methods. MR-Egger regression can provide reliable estimates while adjusting for pleiotropy, even when all instruments are invalid (35). The weighted median assumes that at least 50% of the instruments are valid (36). The weighted mode estimation method is effective in detecting causal effects, showing less deviation and a lower Type I error rate than the MR–Egger regression when the Instrument Strength Independent of Direct Effect (InSIDE) assumption is not met (37). Although the simple mode is not as robust as IVW, it is resistant to pleiotropy (38). We used the Cochran Q test to detect the presence of heterogeneity. A Cochran-Q derived P-value less than 0.05 suggests the presence of heterogeneity (39). The assessment of horizontal pleiotropy is based on Egger intercepts (35). Finally, to identify influential points that affect the pooled IVW estimates, we performed a Leave-one-out (LOO) analysis (40).

In our IVW analysis, we employed the Bonferroni correction (41). For the primary analysis, a *P* value less than 4×10^−4^(adjusted for Bonferroni) was deemed statistically significant, while a *P* value between 0.05 and 4×10^−4^ was considered indicative. For the secondary analysis, we interpreted a *P* value less than 2×10^−4^(adjusted for Bonferroni) as statistically significant and a *P* value between 0.05 and 2×10^−4^ as suggestive.

We conducted all Mendelian Randomization analyses and tests utilizing the “TwoSampleMR”, “ggplot2”, and “meta” packages in R software (version 4.3.1) and the LD Score Regression (LDSC) software (version 1.0.1).

## Results

### Primary analyses

**Figure 2:**
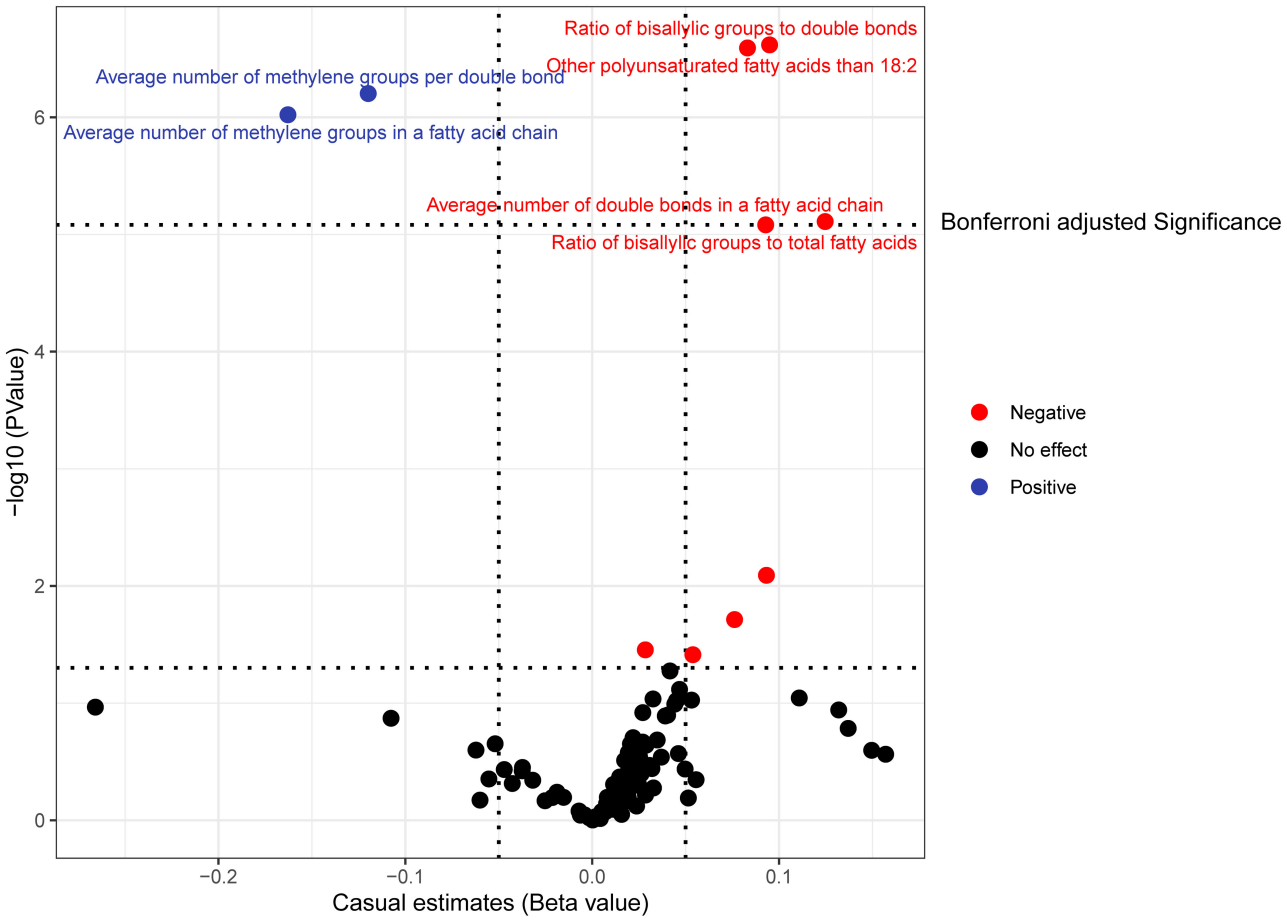
In the primary analysis, the volcano plot indicates the causal relationship between metabolic traits and basal cell carcinoma, using the inverse-variance weighted method.

**Figure 3:**
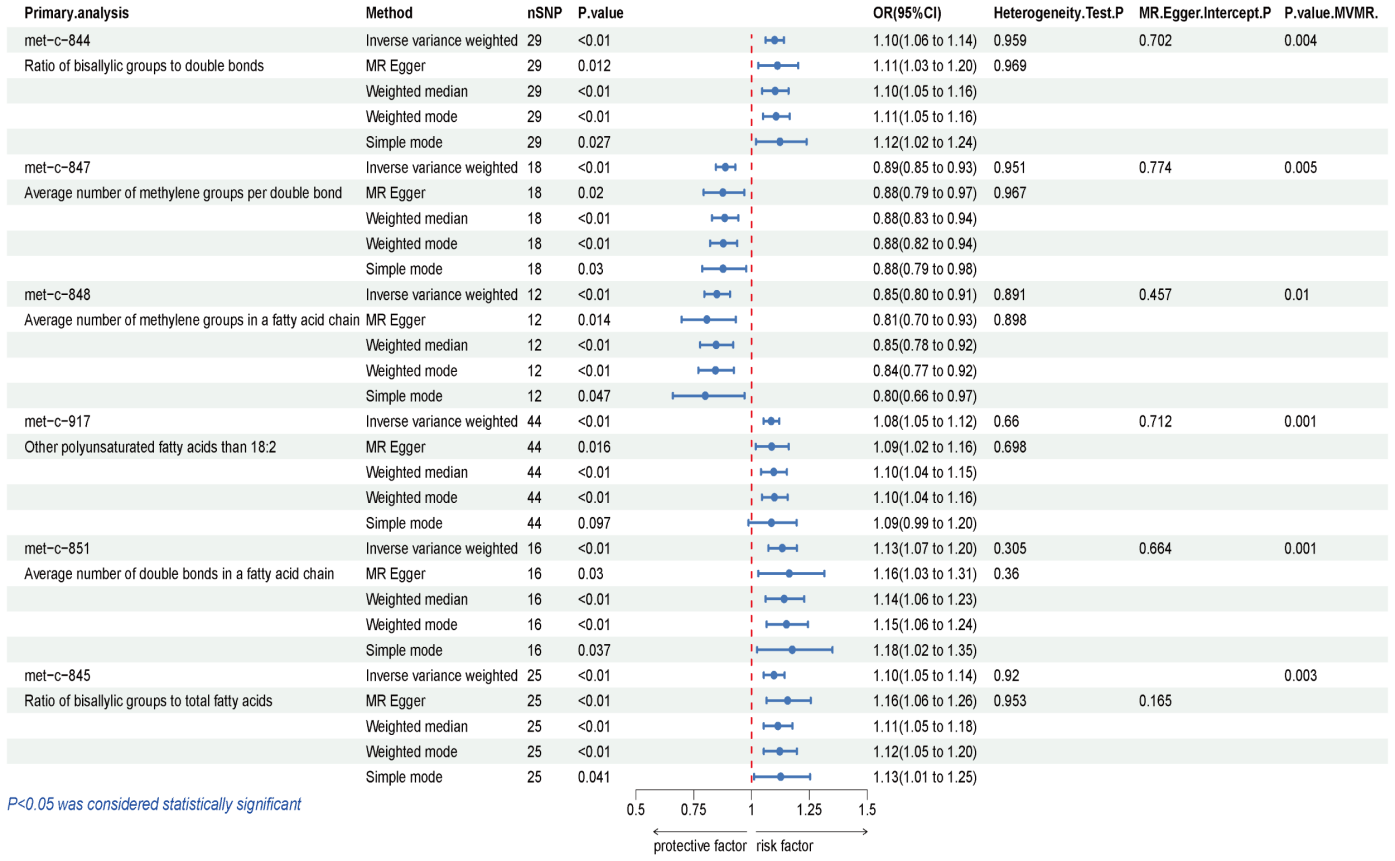
Results of IVW, Sensitivity analysis, Heterogeneity analysis, and MVMR for the causal association between blood metabolites that remain significant after Bonferroni correction in the primary study, IVW, inverse-variance weighted; MVMR, Multivariable Mendelian Randomization.

The causal influence of 123 metabolic traits in circulation on the risk of BCC was assessed in our primary analysis. After strictly controlling the quality of IVs, the MR study finally captured 118 metabolites. Some metabolites were discarded because they could not obtain enough SNPs. The filtered IVs contained 2 to 72 SNPs (3-hydroxybutyrate contains 1 SNP; Glycoproteins produce the most genetic proxies: 72 SNPs). All F-statistics of SNPs related to metabolites are greater than 10, indicating that IVs have strong power. After Bonferroni correction, 6 out of 123 traits showed statistical significance. Notably, these six traits are all ratio indicators related to the degree of fatty acid unsaturation or unsaturation, and 4 of them are associated with the degree of fatty acid unsaturation (**Figures 2**, **3**). Specifically, biomarkers indicating a higher degree of unsaturation, including the ratio of bisallylic groups to double bonds (OR=1.099, 95% confidence interval (CI) 1.060–1.139, *P* =2.41×10^-7^), polyunsaturated fatty acids other than 18:2 (OR=1.085, 95%CI 1.051–1.120, *P* =3.75×10^-7^), the average number of double bonds in a fatty acid chain (OR=1.132, 95%CI 1.072–1.196, *P* =7.76×10^-6^), and the ratio of bisallylic groups to total fatty acids (OR=1.097, 95%CI 1.053–1.142, *P* =8.12×10^-6^), significantly increase the risk of BCC.

In contrast, traits leading to a decrease in the level of unsaturation, including the average number of methylene groups per double bond (OR=0.887, 95%CI 0.846–0.929, *P* =6.19×10^-7^) and the average number of methylene groups in a fatty acid chain (OR=0.849, 95%CI 0.796–0.906, *P* =9.52×10^-7^), are negatively correlated with BCC susceptibility. In all sensitivity analyses, a consistent direction was observed (**Additional File 1**). **Additional Files 2**, **3** display the presence of horizontal pleiotropy and heterogeneity in causal estimates. Detailed information regarding the utilized SNPs is available in **Additional File 4**. LOO analysis did not identify any SNPs likely to bias pooled effect estimates (**Additional File 5**).

### Secondary analyses

**Figure 4:**
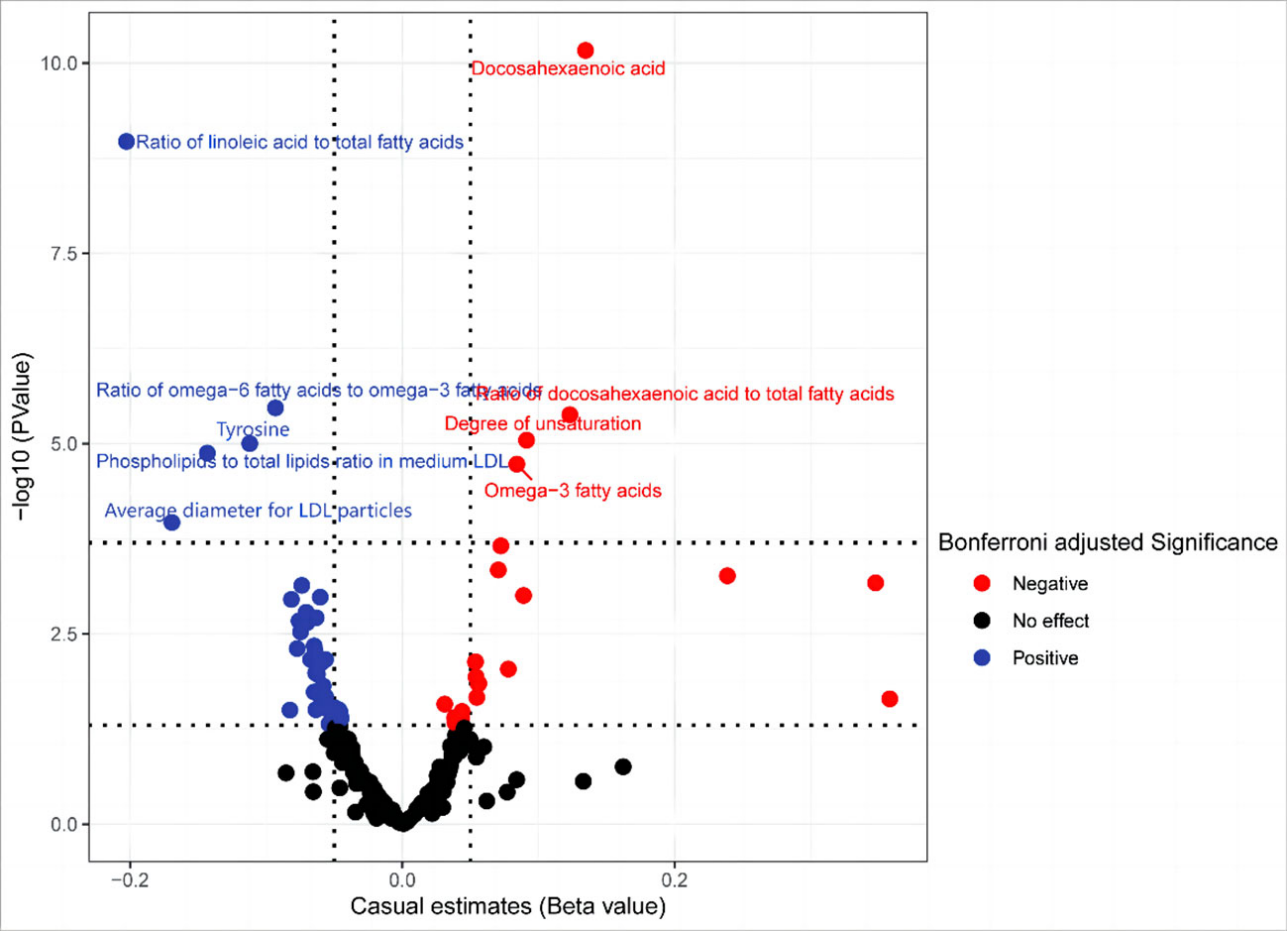
In the secondary analysis, the volcano plot indicates the causal relationship between metabolic traits and basal cell carcinoma using the inverse-variance weighted method.

**Figure 5:**
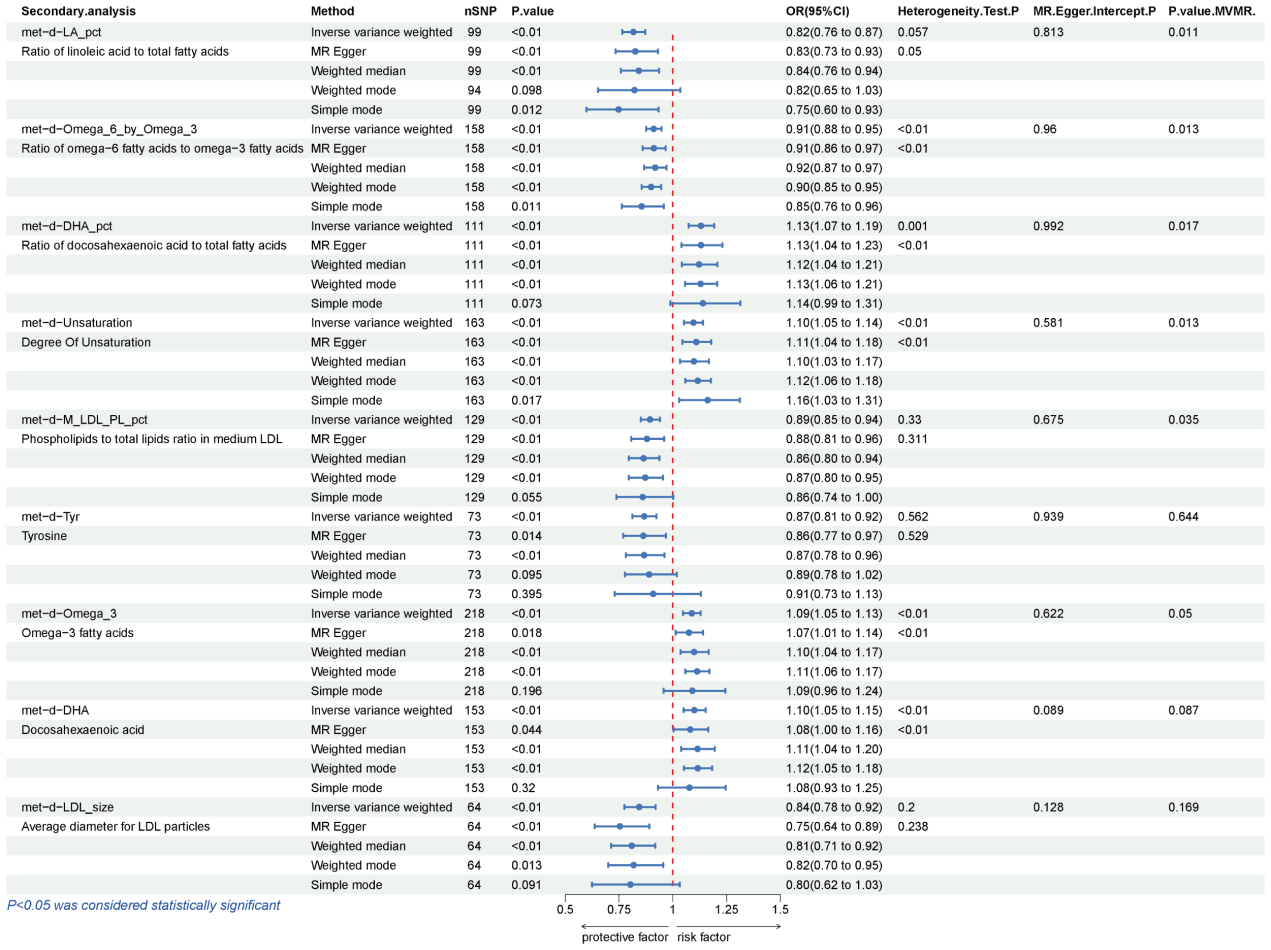
Results of IVW, Sensitivity analysis, Heterogeneity analysis, and MVMR for the causal association between blood metabolites that remain significant after Bonferroni correction in the secondary study, IVW, inverse-variance weighted; MVMR, Multivariable Mendelian Randomization.

Our secondary analysis evaluated the causal effect of 249 metabolic traits on BCC risk. After strictly controlling the quality of IVs, the MR study finally captured 249 metabolites. The filtered IVs contain 9 to 315 SNPs (Acetoacetate contains 9 SNPs; Cholesterol in large HDL produces the most genetic proxies: 330 SNPs). Metabolite-related SNPs have greater than 10 F-statistics. The findings revealed that, among the 249 traits examined, nine serum metabolites maintained statistical significance following Bonferroni correction (**Figure 4**). After the sensitivity and pleiotropy analysis, these nine serum metabolites exhibited significance (**Figure 5**). Among them, the ones associated with an increased incidence of BCC were Ratio of docosahexaenoic acid to total fatty acids (OR= 1.130, 95%CI 1.073–1.191, *P* =4.17×10^-6^), Degree of unsaturation (OR=1.095, 95%CI 1.052–1.140, *P* =9.01×10^-6^), omega-3 fatty acids (OR=1.087, 95%CI 1.046- 1.130, *P* =1.86×10^-5^), Docosahexaenoic acid(OR= 1.100, 95%CI 1.049–1.153, *P* =6.39×10^-5^) and Ratio of omega-3 fatty acids to total fatty acids(OR= 1.074, 95%CI 1.034–1.116, *P* =6.21×10^-4^). On the other hand, the ones associated with a decreased incidence of BCC were Ratio of linoleic acid to total fatty acids (OR=0.816, 95%CI 0.765–0.871, *P* =1.07×10^-9^), Ratio of omega-6 fatty acids to omega-3 fatty acids (OR=0.910, 95%CI 0.875–0.947, *P* =2.39×10^-6^), Phospholipids to total lipids ratio in medium LDL (OR= 0.893, 95%CI 0.850–0.939, *P* =9.98×10^-6^), Tyrosine (OR= 0.866, 95%CI 0.812–0.924, *P* =1.33×10^-5^), Average diameter for LDL particles (OR= 0.844, 95%CI 0.774–0.919, *P* =1.08×10^-4^). In all sensitivity analyses, a consistent direction was observed (**Additional File 6**). **Additional Files 7**, **8** display the presence of horizontal pleiotropy and heterogeneity in causal estimates. Detailed information regarding the utilized SNPs is available in **Additional File 9**. Also, no high-impact SNPs were identified in the LOO analysis that might influence pooled effect estimates (**Additional File 10**).

### Genetic correlation and direction validation

#### LDSC and Steiger

We used LDSC to estimate the genetic correlation between BCC and 15 identified traits. Most showed no evidence of genetic correlation (r_g_ P > 0.05), indicating that the MR results were not confounded by shared genetic factors. However, we observed a possible genetic correlation between BCC and the Ratio of docosahexaenoic acid to total fatty acids (r_g_ = 0.088, se = 0.041, P = 0.033) and Ratio of linoleic acid to total fatty acids (r_g_=0.102, se=0.043, P =0.016) and BCC. This suggests genetic correlation, which measures shared genetic influences between traits. A positive correlation indicates that the same genetic variants affect both traits similarly, implying horizontal pleiotropy — where one variant influences multiple traits through different pathways. This can challenge MR assumptions and lead to misleading causal inferences (29, 30). Consequently, because of their false-positive potential, we need to be more cautious about their results. We also calculated the SNP-heritability of the metabolites using LDSC. The SNP-heritability (the proportion of variance explained by genome-wide SNPs) varied from 0.0215 (Average number of methylene groups in a fatty acid chain) to 0.1713 (Ratio of bisallylic groups to double bonds) (**Additional File 11**). A Steiger test was conducted to validate that the effect direction was from metabolites to BCC. The P values of the Steiger test indicated there wasn’t reverse causation between the traits for the previously mentioned SNPs.

#### Replication analysis and meta-analysis

**Figure 6:**
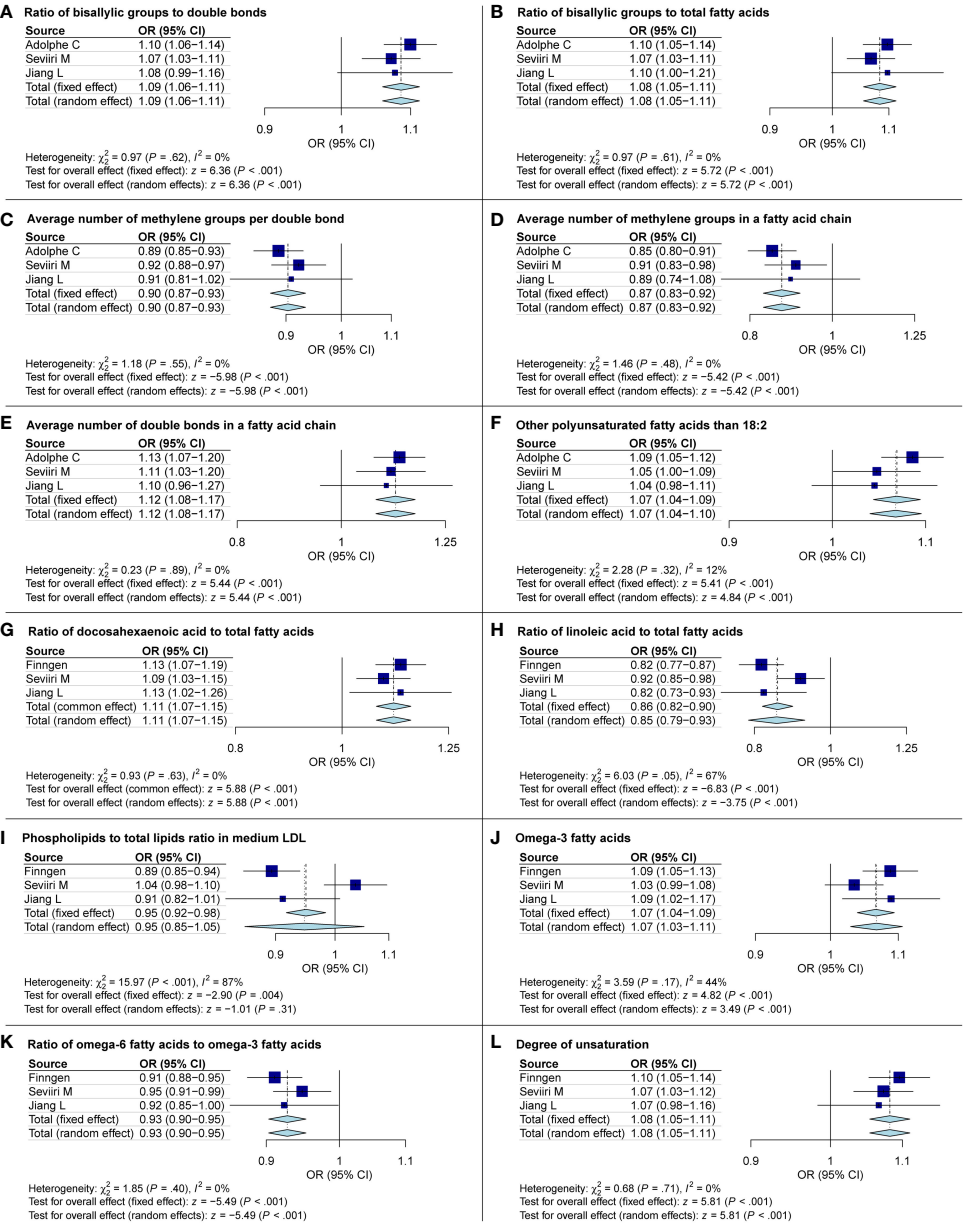
Meta-analysis exploring the causal links between basal cell carcinoma and metabolites. OR, odds ratio; CI, confidence interval.

The IVW results from the discovery group, Seviiri M, and Jiang L datasets were used for replication analysis and meta-analysis to validate our findings further. As anticipated, we noticed analogous trends in the candidate metabolites when compared to the validation group. This lends credence to our assertion that our research outcomes are not mere coincidences. It is noteworthy that the Phospholipids to total lipids ratio in medium LDL from subsequent discussions became insignificant in the meta-analysis. To maintain the rigor of our research, we have decided to exclude it from subsequent discussions (**Figure 6**).

#### Multivariate Mendelian randomization analysis

MVMR was used to estimate causal relationships between candidate traits and BCC risk. From previous studies, we identified three known risk factors associated with an increased risk of BCC: ease of skin tanning, telomere length, and radiation-related disorders (42). After adjusting for these BCC-related risk factors, Our MVMR results indicated that, except for the ratio of linoleic acid to total fatty acids, docosahexaenoic acid, average diameter for LDL particles, and tyrosine, all the other candidate traits could independently influence the occurrence of BCC. These four traits may be associated with BCC, but this association might be masked by other possible risk factors. (**Additional File 12**)

#### Unsaturated fatty acids

**Figure 7:**
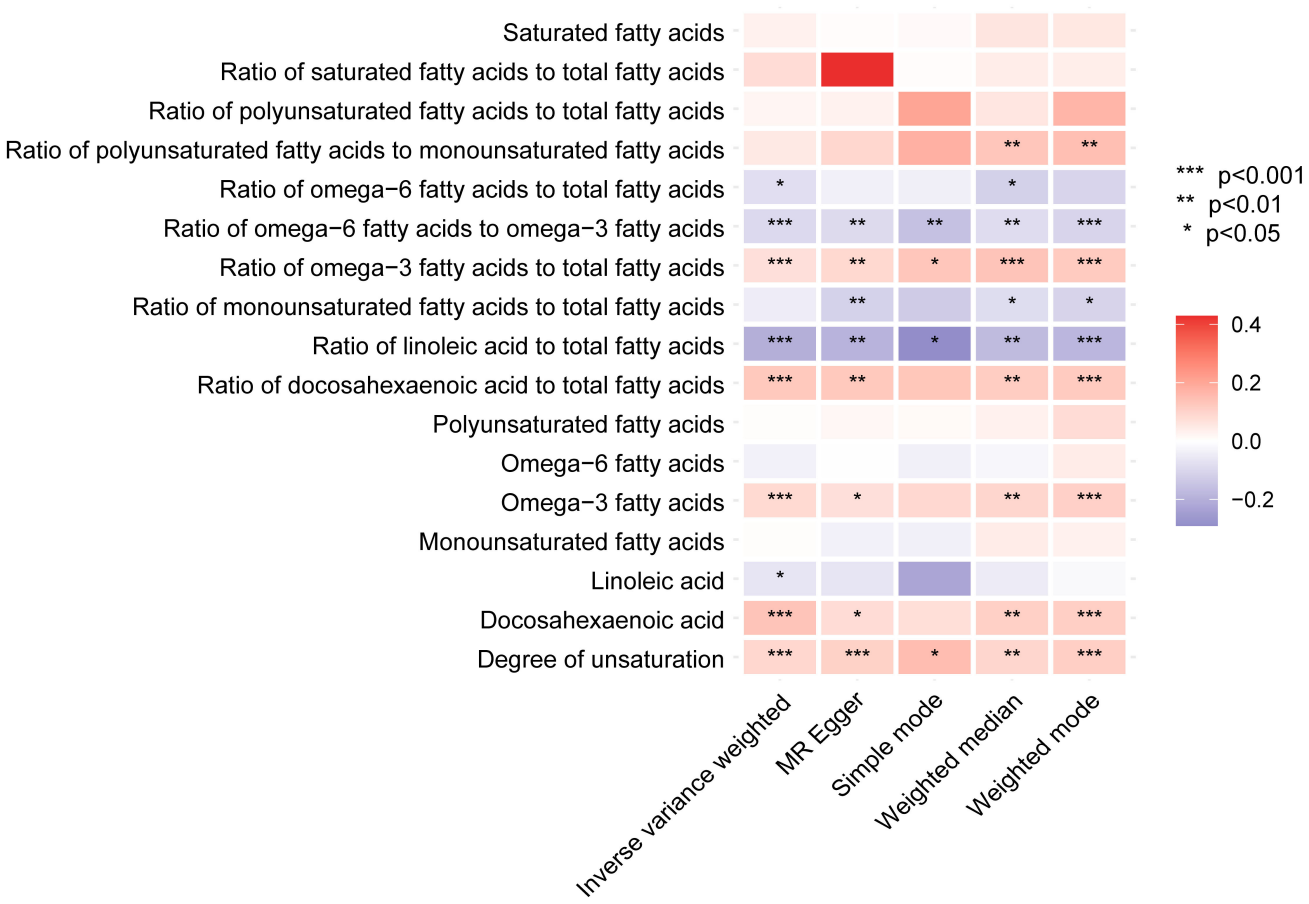
The heatmap displays the influence of fatty acid saturation-related biomarkers on basal cell carcinoma in the secondary analyses based on various methods (IVW, MR-Egger, simple mode, weighted median, and weighted mode). IVW, inverse-variance weighted.

After a thorough analysis, specific metabolites previously identified have been excluded. It was found that the remaining metabolites are linked to unsaturated fatty acids and their level of unsaturation. To understand the correlation between different metabolic characteristics and BCC, a total of 249 metabolic traits in secondary were categorized into nine main groups (**Additional File 13**, **Supplementary Figures S1**-**S8**) (15). Our exploration of these biomarker sets reveals that many genes related to unsaturated fatty acid saturation are positively or negatively associated with BCC risk. Among the 17 features related to unsaturation, 9 exhibit significant or suggestive causal relationships. Although some traits did not yield significant results in sensitivity analysis, their direction of association with unsaturation remains consistent. Specifically, we find that higher unsaturation levels, as known biomarkers, are associated with an increased risk of BCC. The causal heatmap depicting the relationship between unsaturated fatty acids and BCC is shown in **Figure 7**.

## Discussion

In our primary study, MR analysis demonstrated a causal relationship between traits associated with the degree of unsaturation and BCC. Secondary study findings indicated that blood metabolites, particularly those related to PUFAs, are associated with BCC. Both studies employed stringent Bonferroni correction, and their results remained consistent despite originating from different regions and databases. Further analyses (including replication group analysis, meta-analysis, MVMR, Steiger test, and LDSC) identified specific metabolites associated with BCC, such as omega-3 PUFA, DHA, the omega-6/omega-3 PUFA ratio, and the degree of unsaturation. These metabolites all belong to the PUFA category of unsaturated fatty acids. Some lipid-related metabolites were associated with BCC, but caution is needed due to potential confounding factors and common genetic influences that might result in false positives. Further analysis is recommended for a comprehensive evaluation.

Fatty acids are classified into saturated fatty acids (SFAs), monounsaturated fatty acids (MUFAs), and PUFAs, distinguished by their carbon chain structures and degree of hydrogen saturation. SFAs lack double bonds between carbon atoms, making them fully saturated with hydrogen atoms. In contrast, MUFAs contain one double bond, causing a kink in the carbon chain, while PUFAs contain two or more double bonds. The degree of unsaturation, indicated by the number of double bonds in the fatty acid chain, is zero for SFAs, one for MUFAs, and two or more for PUFAs (43). Numerous studies have identified a close relationship between fatty acids and human health, particularly highlighting the role of PUFAs in various diseases (44). Dietary modifications, such as increased fish oil intake, are linked to reduced cardiovascular disease risk because fish and fish oil are rich in Omega-3 fatty acids. Our research focuses on the association between PUFAs and BCC (45). This could support future dietary interventions with PUFAs to prevent BCC. While UV exposure is the main risk factor for BCC, our study found significant metabolites even after adjusting for factors like skin tanning, telomere length, and radiation-related disorders through MVMR analysis. This suggests that PUFAs might be an independent risk factor for BCC. Given BCC’s prevalence, even small preventive measures could have huge benefits for public health.

Previous research has established a connection between the unsaturation level of dietary fats, particularly PUFAs, and BCC (46–49). However, the investigation into the impact of PUFAs and their degree of unsaturation on BCC is still in its early stages. Some studies suggest that a higher degree of fat unsaturation could lead to the generation of free radicals and oxidative stress due to lipid peroxidation, thereby facilitating the development of BCC (47, 48, 50, 51). We found that an increase in the ratio of omega-3 PUFA and DHA (docosahexaenoic acid) increases the risk of BCC. Conversely, an increase in omega-6 PUFA to omega-3 PUFA lowers this risk. This discovery aligns with primary studies, as omega-3 PUFAs generally exhibit greater unsaturation than omega-6. DHA, a type of omega-3 fatty acid containing five cis double bonds, is vulnerable to damage from free radicals, creating harmful advanced lipid peroxidation end products (ALEs) (52, 53). These ALEs can harm cell membranes and cause DNA damage when they build up to toxic levels (54, 55). It is important to mention that there is no correlation between the amount of saturated fatty acids and the occurrence of BCC, regardless of the analytical approach employed. The proportion of various types and degrees of unsaturated fatty acids is a critical factor in BCC. To our surprise, the relative proportions of unsaturated PUFAs also significantly impacted BCC in terms of their absolute levels and their ratios with other PUFAS.

While mammals can synthesize saturated and monounsaturated fatty acids, they cannot synthesize PUFAs. This has led to studies on the dietary regulation of PUFAs and their impact on BCC. The focus has been on Omega-3 and Omega-6 fatty acids, but findings have been inconsistent. Some research suggests that Omega-3 acts as a BCC protective factor, while Omega-6 may enhance BCC expression (56). Other studies indicate that omega-3 PUFAs can reduce skin inflammation and potentially prevent skin cancer, even in organ transplant patients (57–59). An Australian study found that both omega-3 and omega-6 PUFAs may reduce keratinocyte skin cancer risk, especially in high-risk individuals (60). However, a systematic review found no association between dietary omega-3 PUFAs and BCC (61). Considering the vulnerability of observational studies to biases like selection and recall, as well as issues of confounding and reverse causation, it’s no surprise that a consistent connection between BCC and PUFAs is often not established. Furthermore, the complexity of PUFAs, particularly the varying degrees of unsaturation in omega-3 PUFAs and omega-6 PUFAs, makes their categorization problematic for studying their effects on BCC. Moreover, designing and implementing randomized controlled trials poses substantial challenges in this context. Thus, employing MR becomes a highly suitable approach. MR can serve as a valuable alternative method, providing reliable evidence and clarifying the causal relationship between exposure and disease susceptibility. It can overcome the difficulties of implementing dietary interventions in RCTs (62, 63). For instance, a 2021 MR analysis of BCC and PUFAs indicated that genetically predicted elevated levels of Linoleic acid (LA) and alpha-linolenic acid (ALA) were linked to a reduced risk of BCC, while Arachidonic acid (AA) and Eicosapentaenoic acid (EPA) were associated with an increased risk (64). Although LA and AA are omega-6 fatty acids, and ALA and EPA are omega-3 fatty acids, BCC’s risk seems to be independent of the classification of PUFAs, according to this study. Moreover, Other MR studies have shown that increased activity of PUFA desaturase—a key enzyme that catalyzes the introduction of double bonds in PUFAs, therefore enhancing their unsaturation—correlates with a higher risk of developing BCC (65). Given the consistency between these findings and our own, we posit that the degree of saturation in PUFAs may significantly influence the progression of BCC.

Firstly, we analyzed the largest scale serum metabolome against BCC, which had never been used before, and found a relationship between unsaturated fatty acids and BCC. We discovered that the higher the degree of unsaturation in these PUFAs, the higher the risk of developing BCC. This has never been reported in previous studies. Secondly, the F-statistic for each SNP exceeded 10, indicating the robustness of the instruments. Moreover, the Steiger test supports the causal direction from exposure to the outcome, and we applied strict Bonferroni correction to the results, all of which ensured the accuracy of our findings. Additionally, we carefully sampled distinct datasets, effectively mitigating population overlap issues. Secondly, across different MR models, consistent directions and similar magnitudes confirm robustness, with no evidence of horizontal pleiotropy using supplementary statistical methods. Next, we adjusted for confounding factors related to increased BCC risk through MVMR and excluded confounded candidate traits. We then replicated and meta-analyzed results using independent GWAS data, yielding consistent effect estimates. Although some replication estimates were not statistically significant, their consistent direction is reassuring. Lastly, we used LDSC to assess IV heritability and genetic correlation, excluding genetically correlated metabolites, for more persuasive MR estimates. The results of the study have been significantly improved by these analyses, but the limitations of the study must be acknowledged. Nevertheless, recognizing the constraints of our research is crucial. Firstly, we have relaxed the SNP selection threshold due to the limited number of genome-wide significant SNPs, which is a commonly employed method. However, it is worth emphasizing that we have conducted a series of rigorous sensitivity analyses to minimize errors as much as possible. Secondly, our study’s insights are significant, but they are limited by our exclusively European cohort. Future research should include diverse populations to validate our findings across ethnic groups. Furthermore, the MR technique presupposes continuous exposure over a lifetime, which may not accurately reflect real-life scenarios. Therefore, more attention may need to be paid to the direction of the causal relationship, and the estimated level should not be overestimated. Additionally, our MR study cannot explore the cellular and molecular mechanisms through which metabolites affect BCC. Moreover, the MR approach is effective for causal inference, but findings should be substantiated through rigorous RCTs to establish causal relationships. Despite challenges in conducting RCTs, focusing on RCTs to explore the impact of PUFA supplements on BCC holds significant public health implications for disease prevention, given that BCC is the most common cancer worldwide.

## Conclusions

In conclusion, our research presents initial evidence underscoring the significant impact of unsaturated fatty acid saturation levels on the progression of BCC. We suggest that modifying one’s diet, specifically focusing on the intake of PUFAs, may be an effective strategy for preventing BCC. However, implementing such measures in a clinical setting would require additional RCTs and molecular experimental validation.

## Data availability statement

The datasets presented in this study can be found in online repositories. The names of the repository/repositories and accession number(s) can be found in the article/**Supplementary material**.

## Author contributions

BW: Funding acquisition, Project administration, Resources, Writing – original draft, Writing – review & editing. XZ: Funding acquisition, Project administration, Resources, Supervision, Writing – original draft, Writing – review & editing. FP: Writing – original draft, Writing – review & editing. QW: Data curation, Investigation, Methodology, Writing – original draft, Writing – review & editing. QL: Writing – original draft, Writing – review & editing. HQ: Writing – original draft, Writing – review & editing. SZ: Writing – original draft, Writing – review & editing.
