# Supplementary figures and images for: Association between blood metabolites and basal cell carcinoma risk: a two-sample Mendelian randomization study
Source: Front Endocrinol (Lausanne). 2024 Jul 9;15:1413777. doi: 10.3389/fendo.2024.1413777 (PMC11263015; doi:10.3389/fendo.2024.1413777)

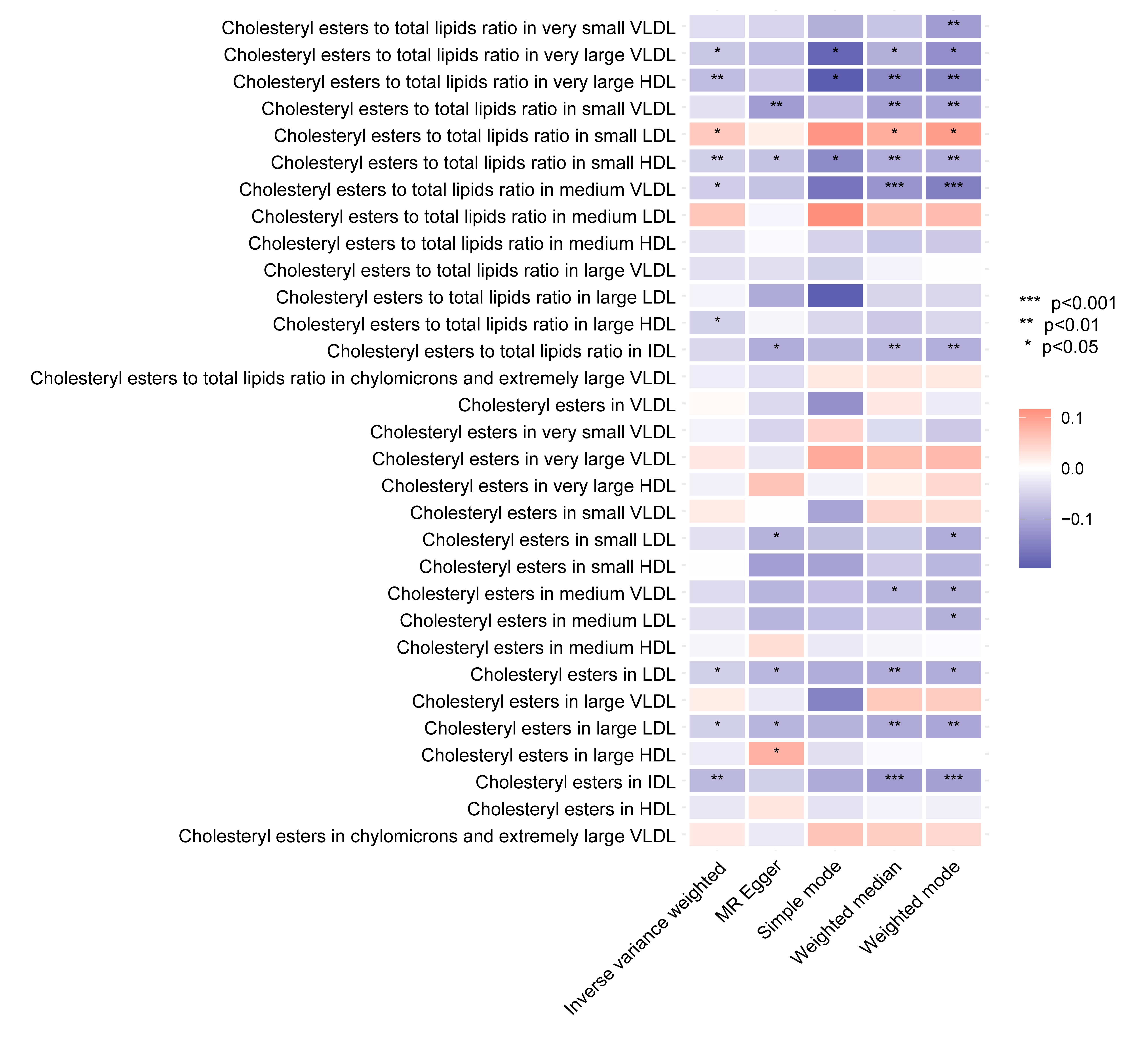

Supplement: Supplementary file 1 [file DataSheet_1.zip › Supplementary Figure 2. cholesterol Ester.tif]

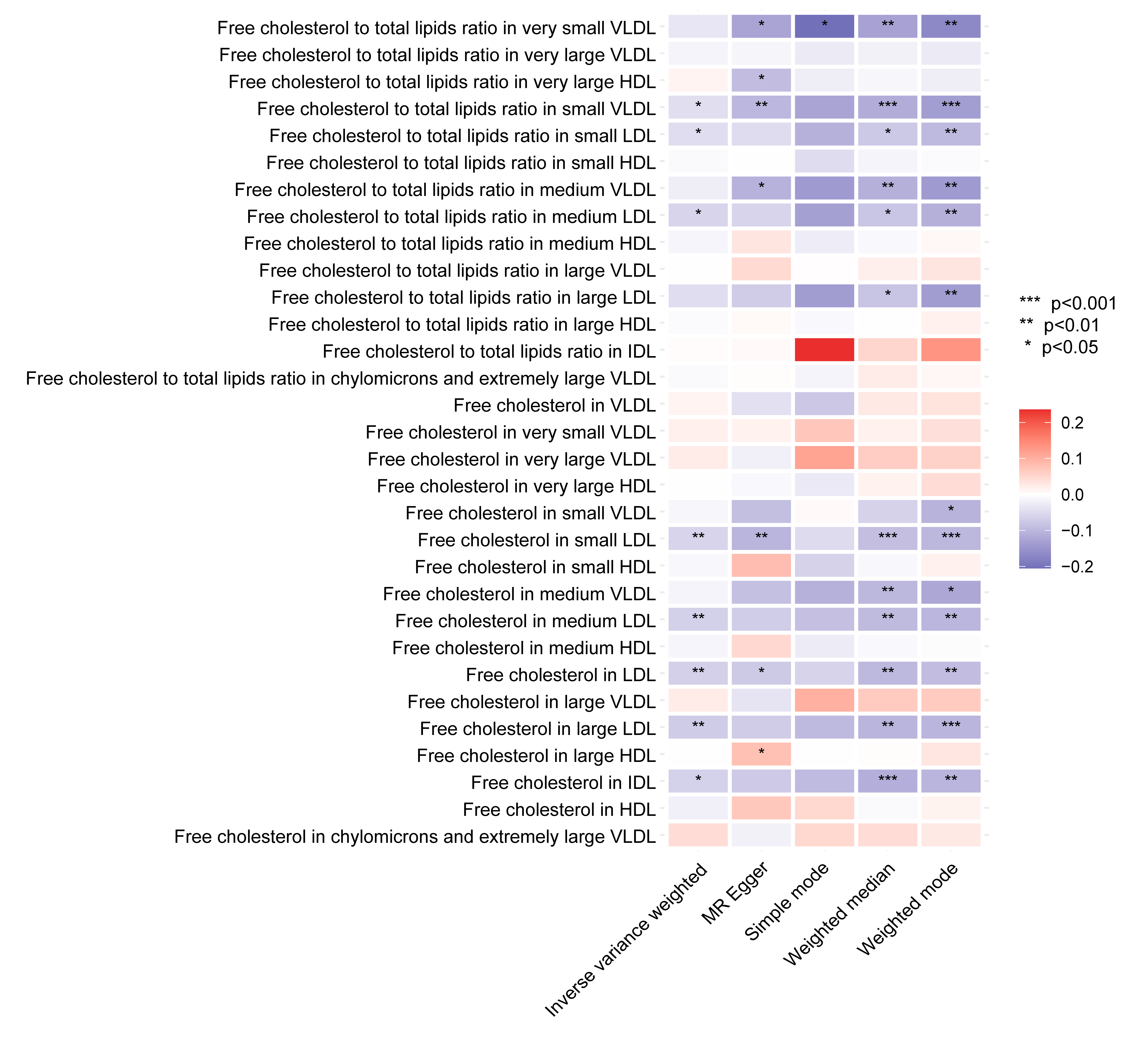

Supplement: Supplementary file 1 [file DataSheet_1.zip › Supplementary Figure 3. Free cholesterol.tif]

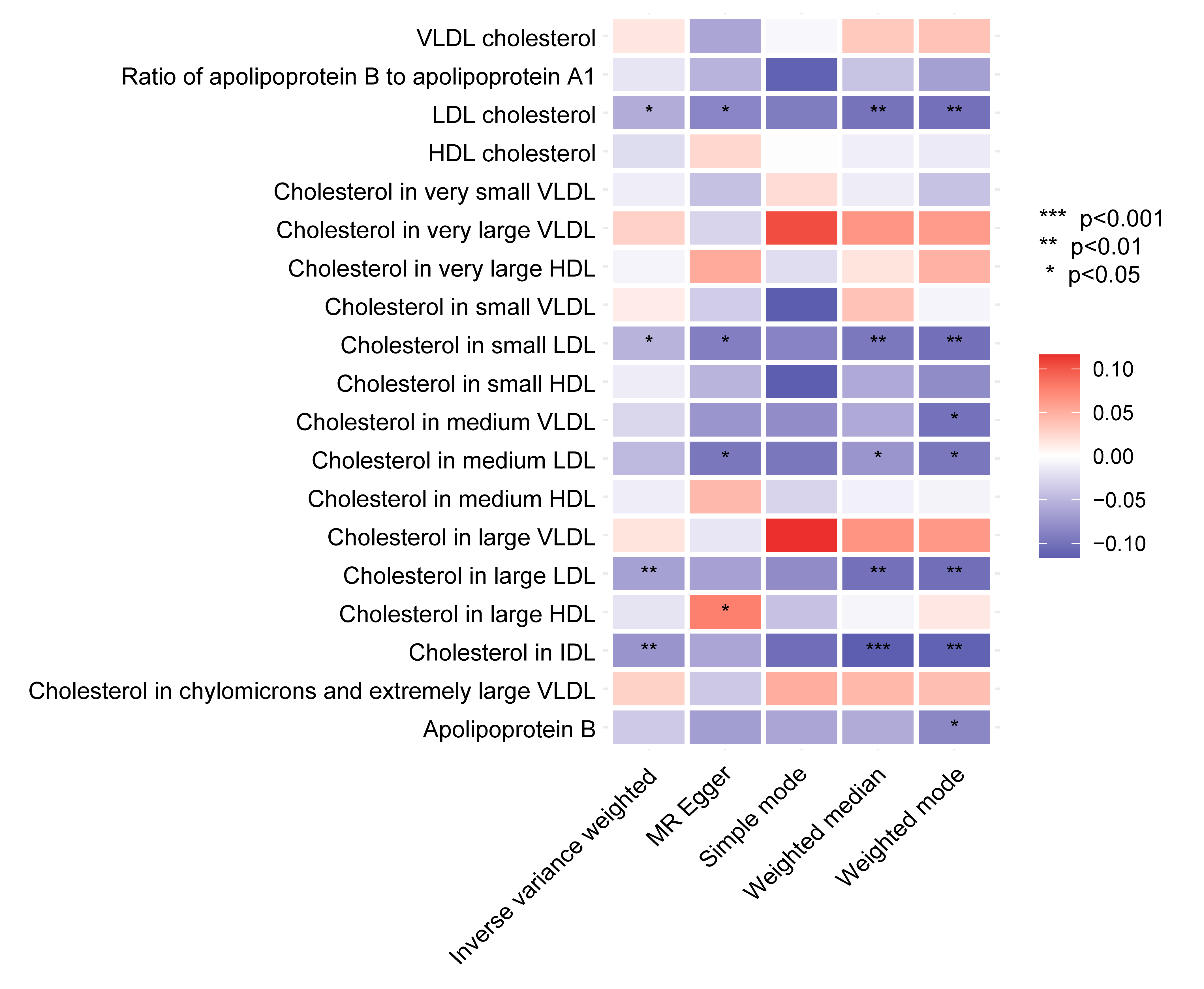

Supplement: Supplementary file 1 [file DataSheet_1.zip › Supplementary Figure 4. lipoprotein_cholesterol.tif]

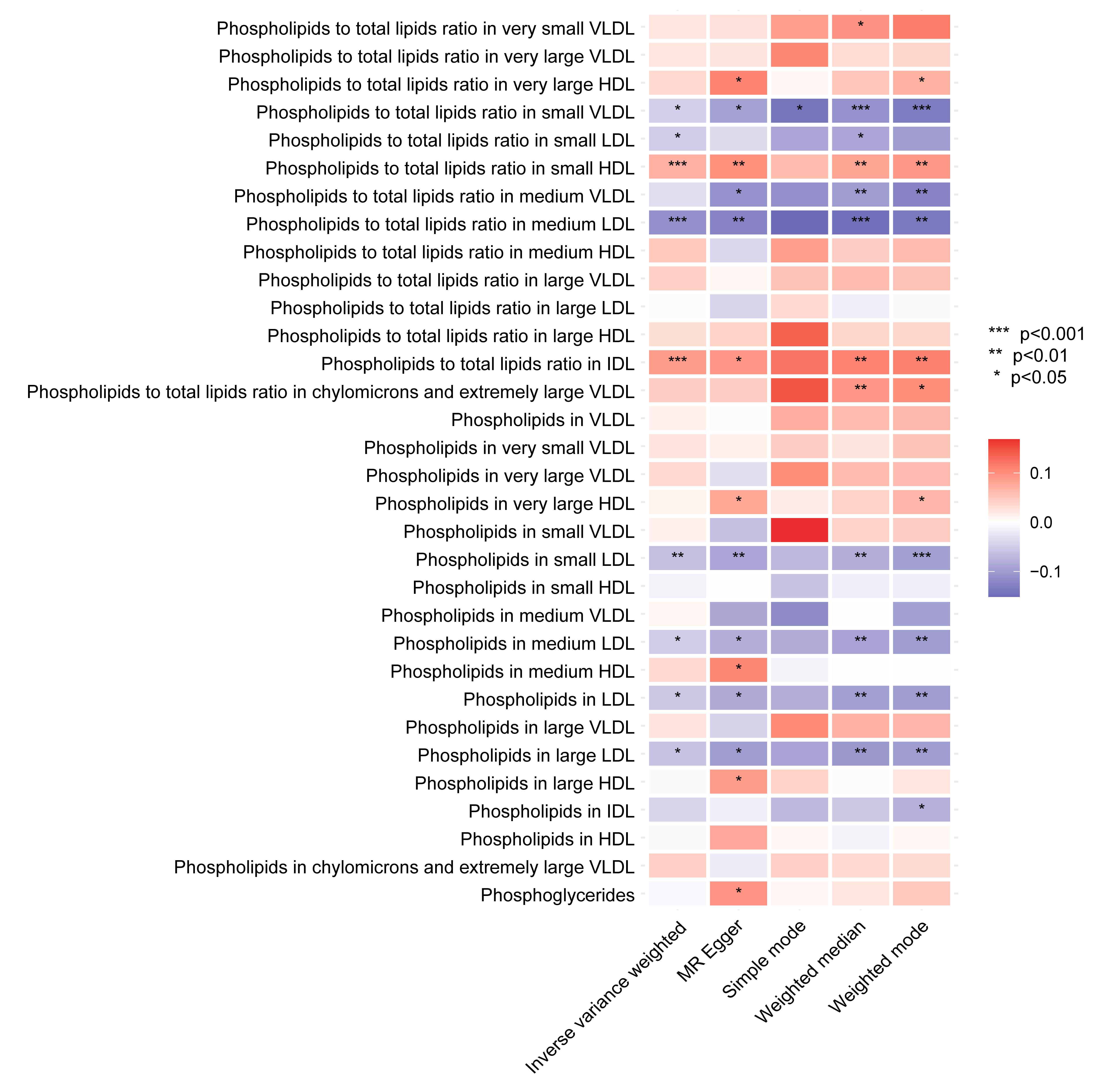

Supplement: Supplementary file 1 [file DataSheet_1.zip › Supplementary Figure 5. Phospholipids.tif]

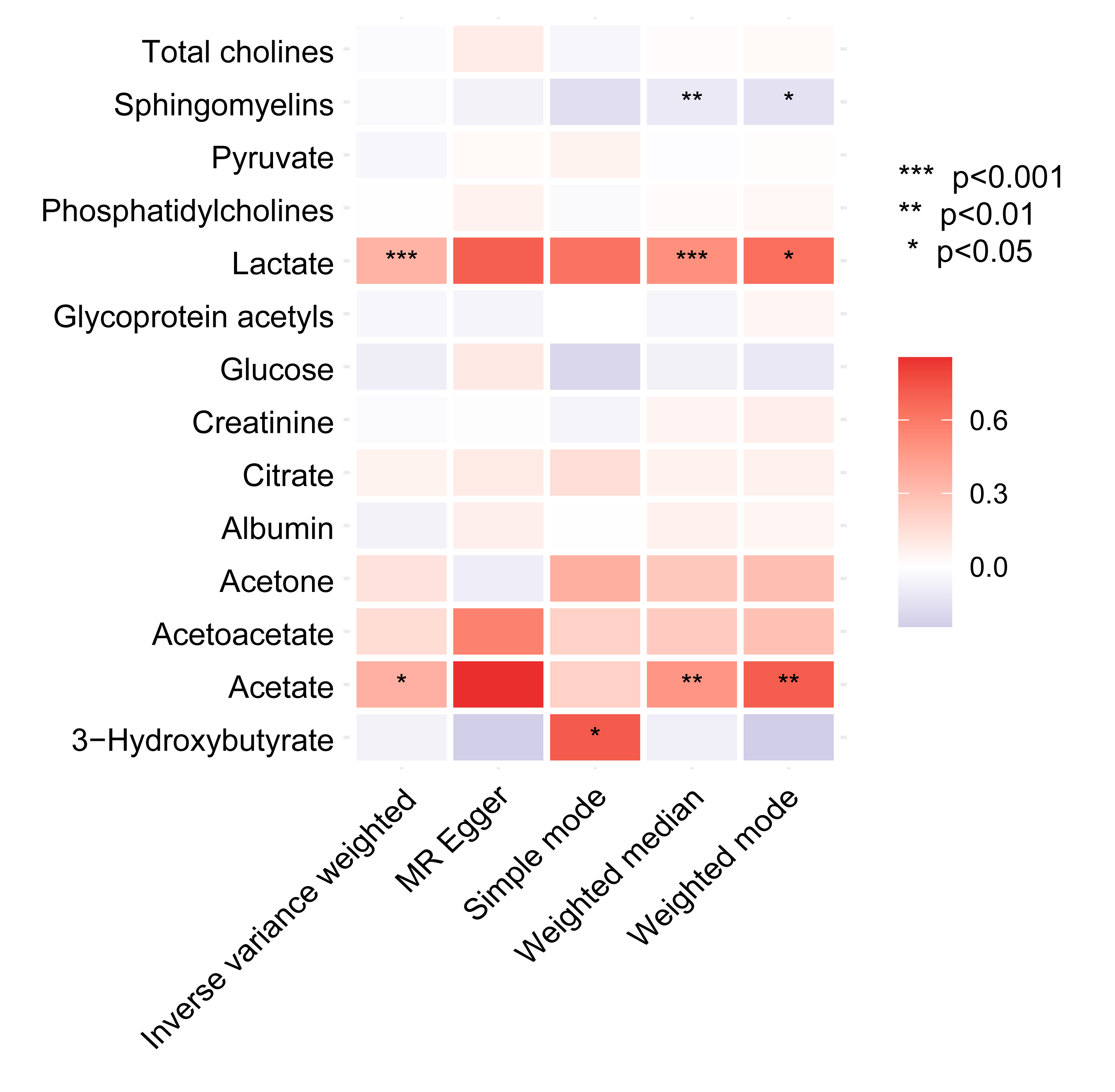

Supplement: Supplementary file 1 [file DataSheet_1.zip › Supplementary Figure 6. metabolite.tif]

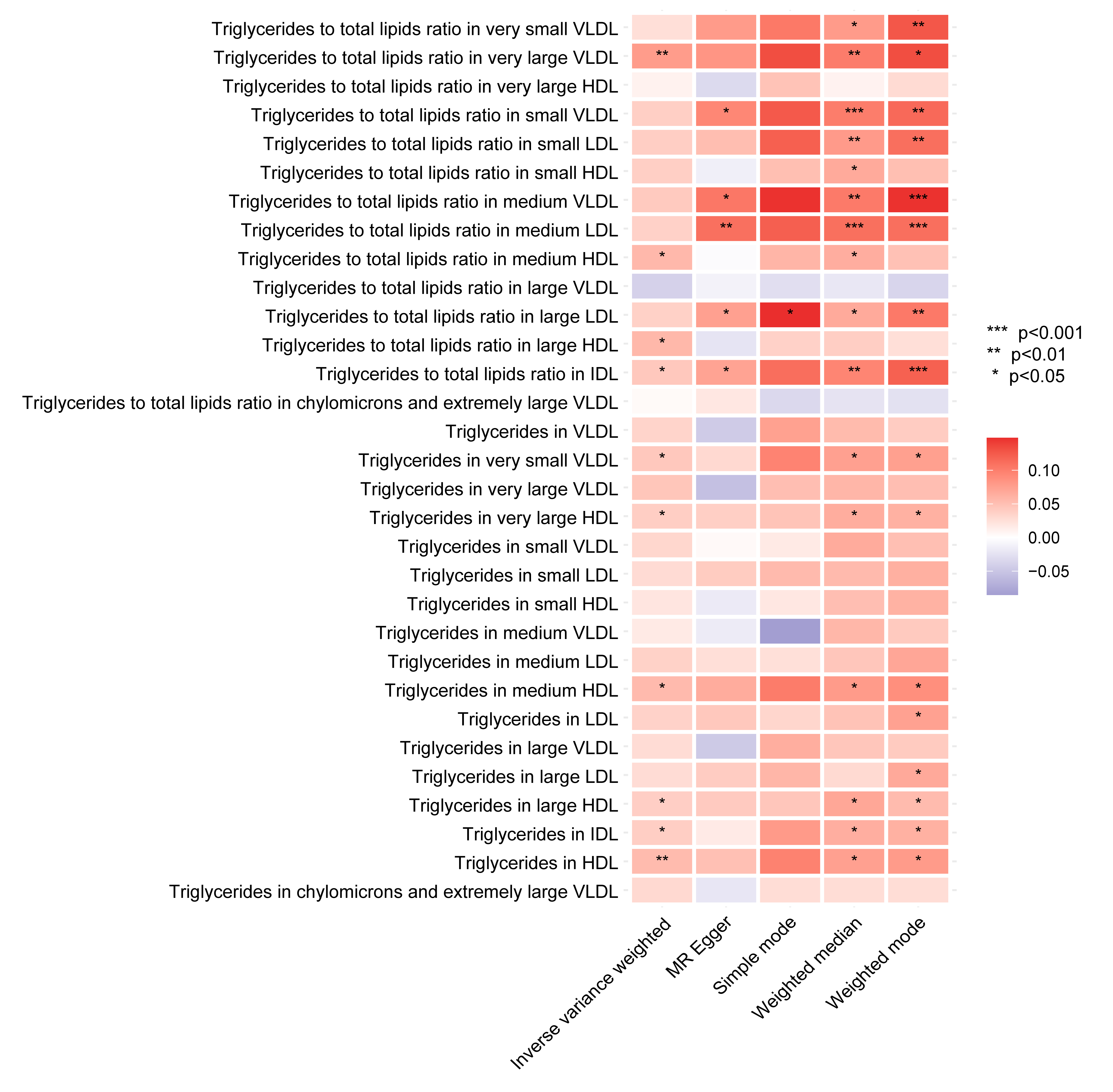

Supplement: Supplementary file 1 [file DataSheet_1.zip › Supplementary Figure 7. Triglycerides.tif]

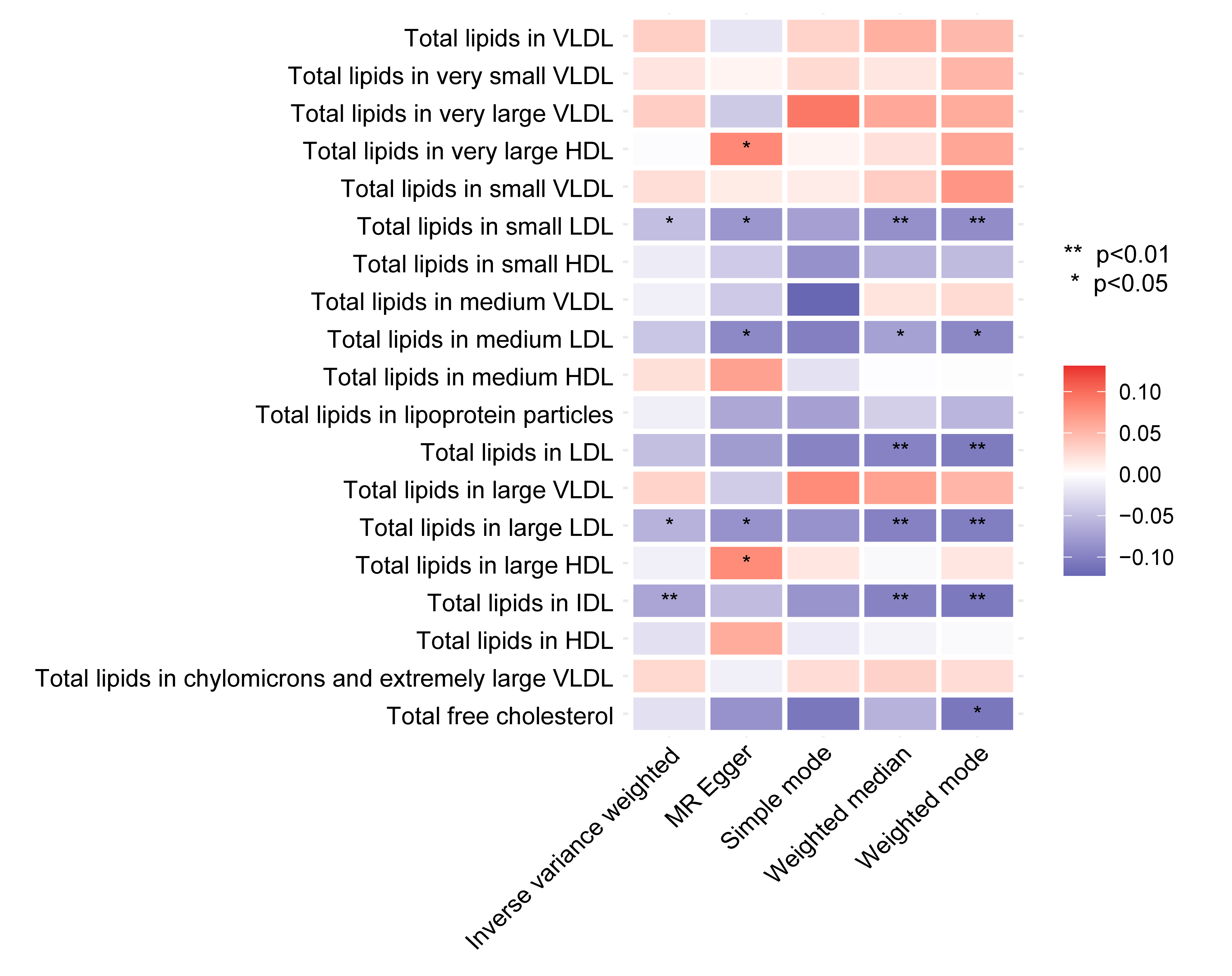

Supplement: Supplementary file 1 [file DataSheet_1.zip › Supplementary Figure 8. total lipid.tif]

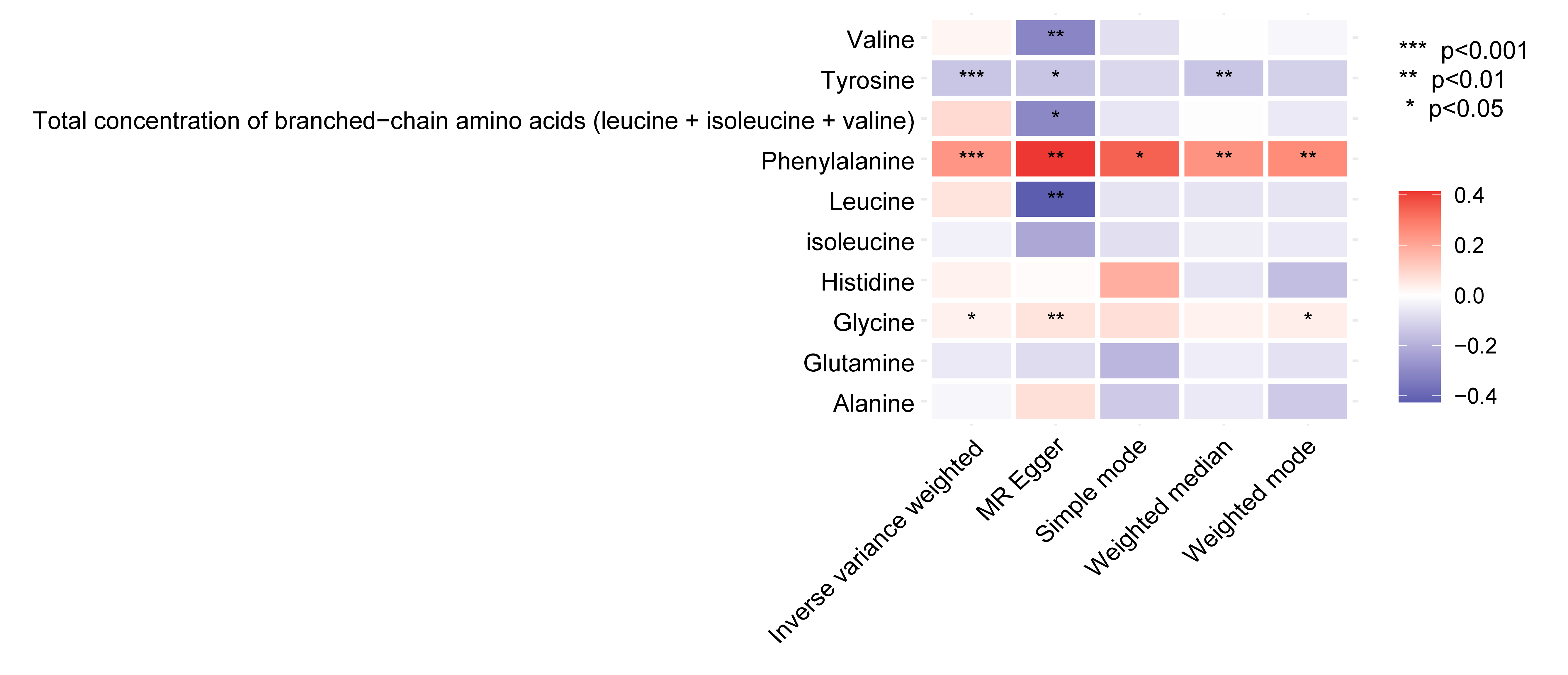

Supplement: Supplementary file 1 [file DataSheet_1.zip › Supplementary Figure 1. Amino acid.tif]
